# Supplementary material for: CD8+ T cell-mediated endotheliopathy is a targetable mechanism of neuro-inflammation in Susac syndrome
Source: Nat Commun. 2019 Dec 18;10:5779. doi: 10.1038/s41467-019-13593-5 (PMC6920411; doi:10.1038/s41467-019-13593-5)
Supplement: Supplementary file 1 — Supplementary Information [file 41467_2019_13593_MOESM1_ESM.pdf]

Supplementary Information

CD8^{+} T cell-mediated endotheliopathy is a targetable mechanism of neuro-inflammation in Susac syndrome, Gross *et al.* 2019

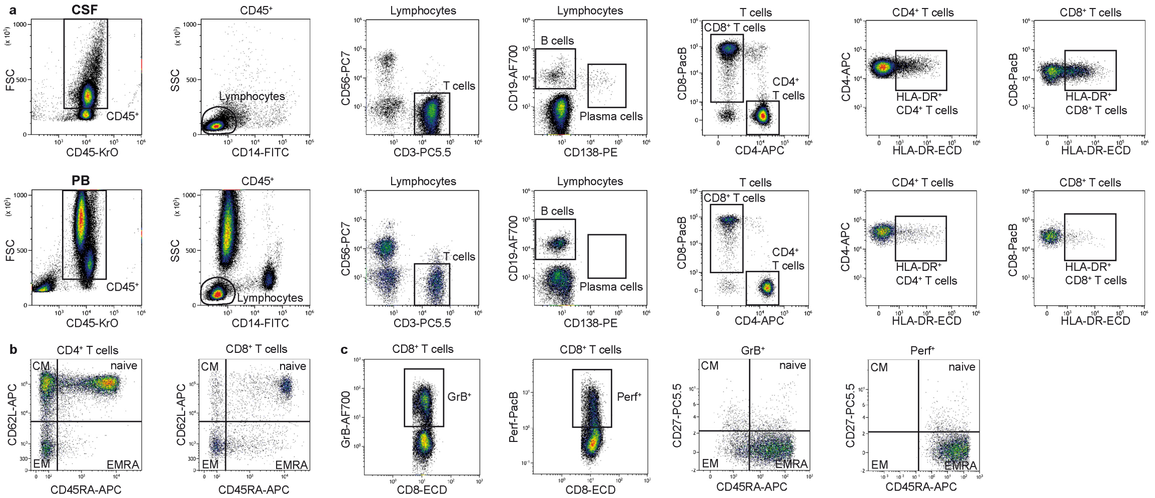

Supplementary Figure 1. Gating strategies identifying immune-cell subsets. **a.** Gating strategy for detection of CD19$^+$ B cells, CD138$^+$ plasma cells, CD4$^+$ T cells, CD8$^+$ T cells CD4$^+$HLADR$^+$ T cells, and CD8$^+$HLADR$^+$ T cells in the cerebrospinal fluid (CSF, top) and peripheral blood (PB, bottom) (Figure 1b, 3a-b, Supplementary Figure 2, 5a, 6). **b.** Gating strategy to detect CD45RA$^+$CD62L$^+$ naïve ($T_{naïve}$), CD45RA$^-$CD62L$^+$ central memory (T_{CM}), CD45RA$^-$CD62L$^-$ effector memory (T_{EM}), and CD45RA$^+$CD62L$^-$ CD45RA re-expressing effector memory (T_{EMRA}) cells constituting CD4$^+$ and CD8$^+$ T cells in the PB (Figure 1c, Supplementary Figure 3, 4a). **c**. Gating strategy to identify proportions of granzyme B (GrB$^+$) and perforin (Perf$^+$) expressing cells within distinct CD8$^+$ T-cell subsets (Supplementary Figure 5b).

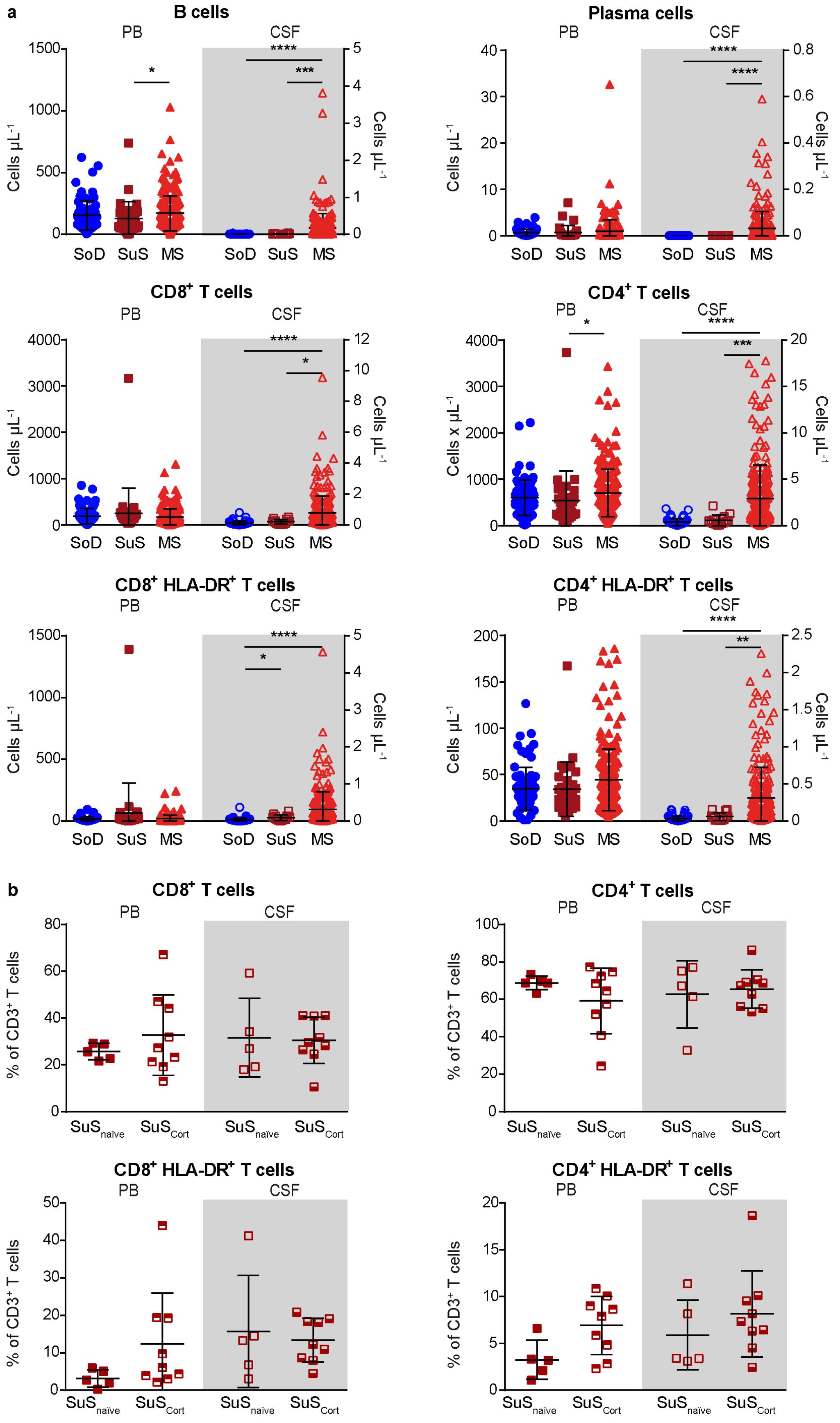
a
B cells
PB
CSF
Cells µL-1
SoD SuS MS SoD SuS MS
Plasma cells
CD8+ T cells
CD4+ T cells
Cells x µL-1
CD8+ HLA-DR+ T cells
CD4+ HLA-DR+ T cells
b
CD8+ T cells
CD4+ T cells
% of CD3+ T cells
SuSnaïve SuSCort SuSnaïve SuSCort
CD8+ HLA-DR+ T cells
CD4+ HLA-DR+ T cells

Supplementary Figure 2. Absolute cell numbers and effect of cortisone. a. Graphs representing total cell numbers of $CD19^+$ B cells (top left), $CD138^+$ plasma cells (top right), $CD8^+$ T cells (middle left), $CD4^+$ T cells (middle right), HLA-DR^+ $CD8^+$ T cells (bottom left), and HLA-DR^+ $CD4^+$ T cells (bottom right) in the peripheral blood (PB; closed symbols, SoD = 75; SuS = 32; MS = 225) and cerebrospinal fluid (CSF; open symbols; SoD = 75; SuS = 14; MS = 228) of somatic symptom disorders (SoD; closed blue circles), SuS (closed cayenne squares), and MS patients (closed red triangles). **b.** Graphs representing the proportions of $CD8^+$ T cells (upper left), $CD4^+$ T cells (top right), HLA-DR^+CD8^+ T cells (bottom left), and HLA-DR^+CD4^+ T cells (bottom right) among $CD3^+$ T cells in the peripheral blood (PB) and cerebrospinal fluid (CSF) of treatment-naïve SuS patients (uniform squares, n = 5) and SuS patients on corticosteroids (divided squares, n = 9) at the time of blood/CSF withdrawal. Statistical analysis was performed using the Kruskal-Wallis test with Dunn’s post-test (a) or Mann-Whitney test (b), respectively. Error bars indicate the mean ± s.d.; *: p <0.05; **: p <0.01; ***: p <0.001; ****: p<0.0001. Source data are provided as a Source Data file.

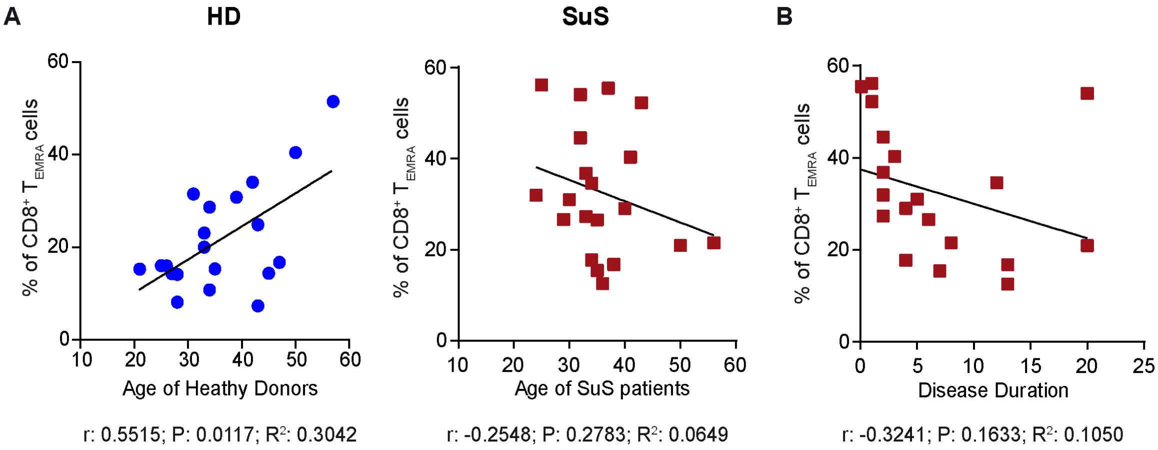

Supplementary Figure 3. Association of demographic features with CD8$^+$ T$_{EMRA}$ cells. a. Correlation of the age of HD (left; closed blue circles, n = 20) and SuS patients (right; closed cayenne squares, n = 20) with the proportion of CD8$^+$ T$_{EMRA}$ cells among peripheral CD8$^+$ T cells. **b.** Correlation of the duration of disease in SuS patients (closed cayenne squares, n = 20) with the proportions of CD8$^+$ T$_{EMRA}$ cells among peripheral CD8$^+$ T cells. Linear regression was analyzed by Pearson test. Source data are provided as a Source Data file.

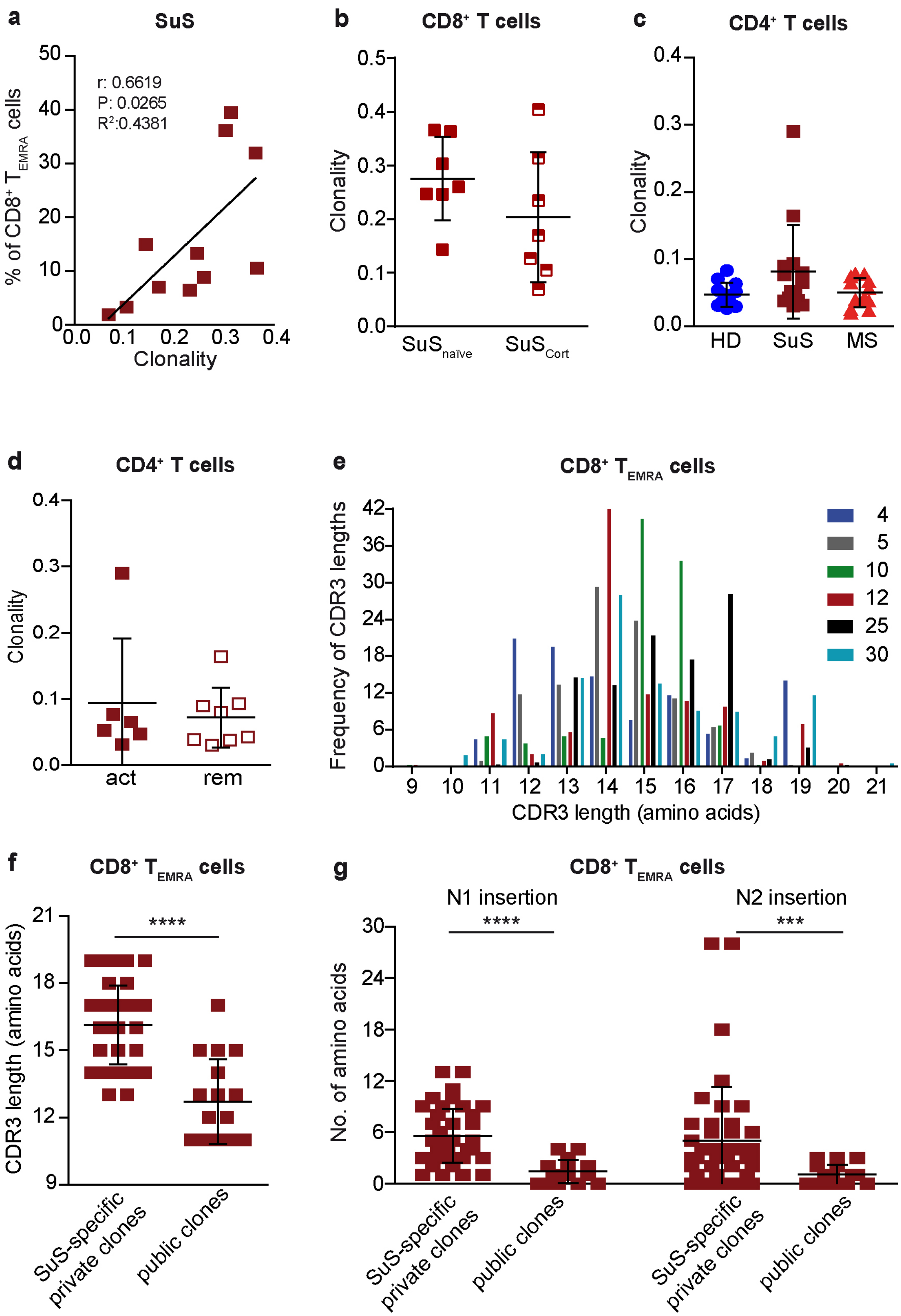
a
SuS
r: 0.6619
P: 0.0265
R²:0.4381
% of CD8⁺ TEMRA cells
Clonality
b
CD8⁺ T cells
Clonality
SuSnaïve
SuSCort
c
CD4⁺ T cells
Clonality
HD
SuS
MS
d
CD4⁺ T cells
Clonality
act
rem
e
CD8⁺ TEMRA cells
Frequency of CDR3 lengths
CDR3 length (amino acids)
4
5
10
12
25
30
f
CD8⁺ TEMRA cells
CDR3 length (amino acids)
****
SuS-specific private clones
public clones
g
CD8⁺ TEMRA cells
N1 insertion
N2 insertion
****
***
No. of amino acids
SuS-specific private clones
public clones
SuS-specific private clones
public clones

Supplementary Figure 4. Clonal characteristics of T-cell repertoires of SuS patients. a. Correlation between repertoire clonality of total CD8$^+$ T cells and the proportion of CD8$^+$ T_{EMRA} cells in the blood of SuS patients (closed cayenne squares, n = 11). **b.** Clonality in the CD8$^+$ T-cell repertoire of treatment-naïve SuS patients (closed cayenne squares, n = 7) and SuS patients on corticosteroids (divided cayenne squares, n = 7). **c.** Clonality of CD4$^+$ T-cell repertoires of HD (closed blue circles, n = 12), SuS patients (closed cayenne squares, n = 14), and MS patients (closed red triangles, n = 12). **d.** Clonality of CD4$^+$ T cell repertoire of SuS patients with clinically active disease (closed cayenne squares, n = 6) or in clinical remission (open cayenne squares, n = 8). **e.** *CDR3* length distribution of CD8$^+$ T_{EMRA} cell repertoire in SuS patients (n = 6). **f.** *CDR3* length of SuS-specific and public clones constituting the ten most prevalent clones in CD8$^+$ T_{EMRA} cell repertoire of 6 SuS patients. **g.** Number of nucleotide insertions in the N1 and N2 regions of the CDR3 of SuS-specific private clones (n = 42 clones) and public clones (n = 17 clones). Linear regression was analyzed using the Pearson test (a). Statistical analysis was performed using the Kruskal-Wallis test with Dunn's post-test (c), unpaired Student t test (f), or Mann-Whitney test (b, d, g), respectively. Error bars indicate the mean ± s.d.; *: $p < 0.05$; **: $p < 0.01$; ***: $p < 0.001$; ****: $p < 0.0001$. Source data are provided as a Source Data file.

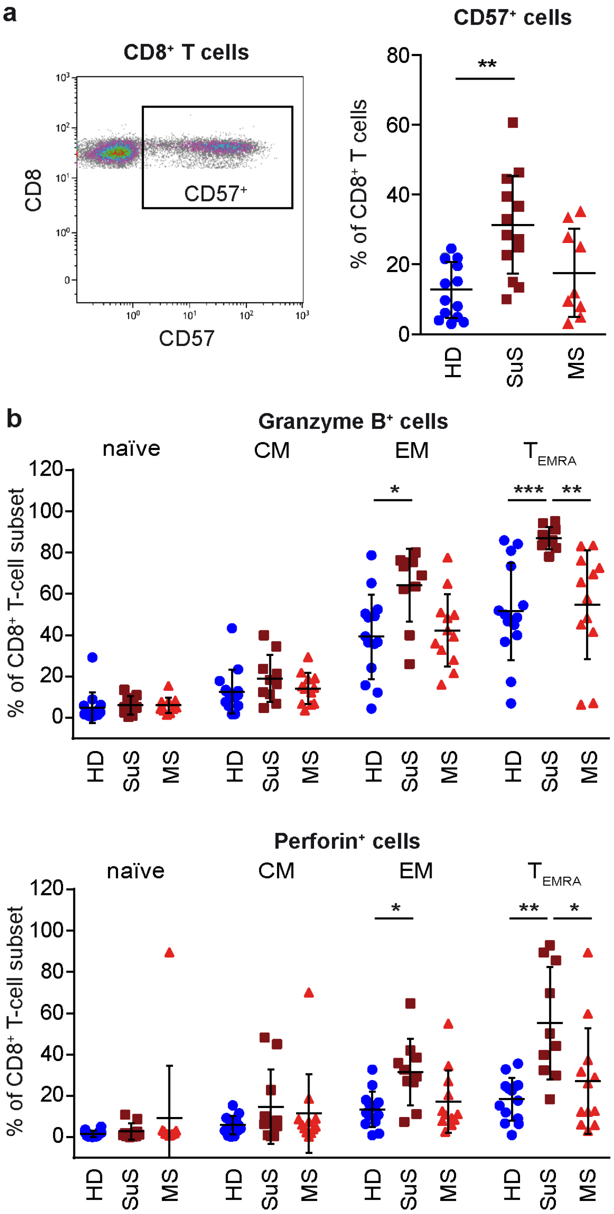

Supplementary Figure 5. Cytotoxic potential of CD8^{+} T cells in SuS. a. Quantification of CD57 expressing CD8^{+} T cells circulating in the blood of HD (closed blue circles, n = 14), SuS patients (cayenne squares, n = 14) and MS patients (red triangles, n = 9). **b.** GrB (top) and perforin (bottom) expression in naïve and memory blood CD8^{+} T-cell subsets in HDs (n = 14), SuS (n = 10) and MS patients (n = 12). Statistical analysis was performed using the Kruskal-Wallis test with Dunn’s post-test. Error bars indicate the mean ± s.d.; *: $p < 0.05$; **: $p < 0.01$; ***: $p < 0.001$. Source data are provided as a Source Data file.

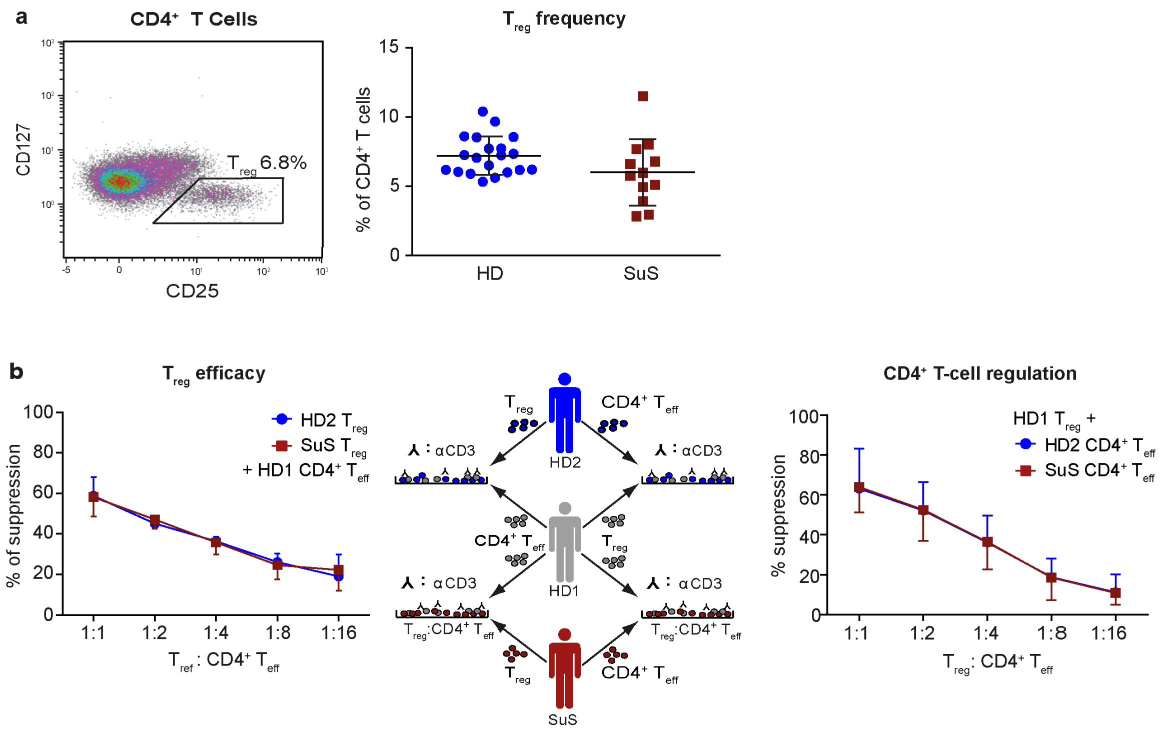

Supplementary Figure 6. Immune regulation in SuS. a. Density plot showing gating strategy and graphs representing proportions of $CD25^{+}CD127^{low}$ regulatory T cells (T_{reg}) in peripheral $CD4^{+}$ T cells of HD (closed blue circles, n = 20) and SuS patients (cayenne squares, n = 12). **b.** Middle: Experimental set-up of allogenic suppression assays to test the suppressive capacity of regulatory T cells. Left: Graphs representing the degree of suppression of proliferation of HD $CD4^{+}$ T cells upon co-culture with titrated numbers of T_{reg} from HD, and SuS (n = 3). Right: Graphs representing the degree of suppression of HD (n = 7) and SuS patient (n = 7) $CD8^{+}$ (middle) and $CD4^{+}$ (bottom) T-cell proliferation upon co-culture with titrated numbers of independent HD T_{reg}. Statistical analysis was performed using the unpaired Student t test (a), or two-way ANOVA with Bonferroni post-test (b), respectively. Error bars indicate the mean ± s.d.; *: $p < 0.05$. Source data are provided as a Source Data file.

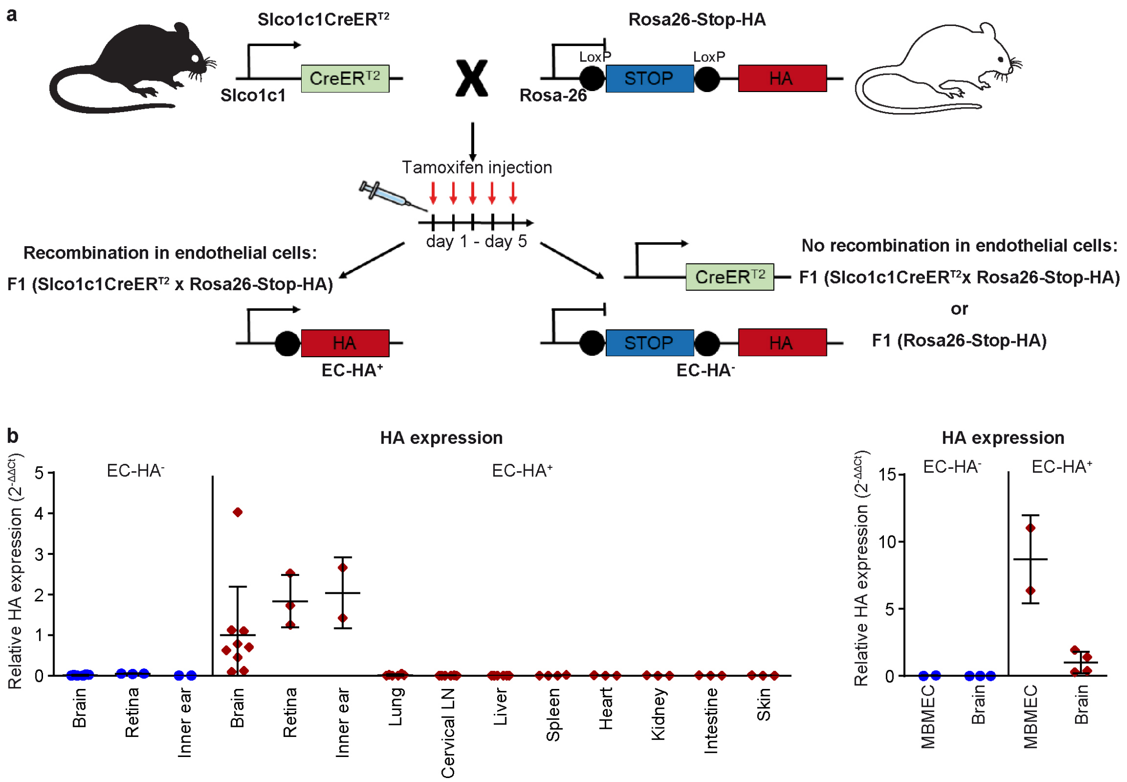

Supplementary Figure 7. Generation of EC-HA⁺ mice. a. Scheme of the transgenic mice used for this study. In the Slco1c1-CreER^{T2} mice, the transgene encodes for the Cre recombinase fused to the ligand-binding domain of the human estrogen receptor (ERT2) under the control of the Slco1c1 promoter. In the Rosa-Stop-HA mice, a LoxP-flanked Stop cassette followed by the *influenza* virus hemagglutinin (HA) sequence was introduced in the ubiquitously active Rosa26 locus by a knock-in approach. In double transgenic mice resulting from the crossing of Rosa-Stop-HA mice with Slco1c1-CreER^{T2} mice, upon tamoxifen injection, Cre-mediated recombination allows transcription of HA in brain microvascular endothelial cells as well as in retina and inner ear. These mice are named EC-HA^{+} mice. In the single transgenic littermate control mice tamoxifen injection does not result in HA expression in brain endothelial cells, retina, and inner ear. These mice are referred to as EC-HA^{-} mice. **b**. Quantification of HA mRNA expression by qRT-PCR in different organs of EC-HA^{+} (closed cayenne diamonds) or EC-HA^{-} (closed blue circles) mice (n = 2-9 per group, left). Detection of HA mRNA expression in mouse brain microvascular endothelial cells (MBMEC) from EC-HA^{-} or EC-HA^{+} mice (right) (MBMEC originated from a pool of 5 mice/group in each of the 2 independent experiments; EC-HA^{-} brain, n = 3), EC-HA^{+} brain, n = 4). The mean HA expression in EC-HA^{+} brain was set to 1. Error bars indicate the mean ± s.d. Source data are provided as a Source Data file.

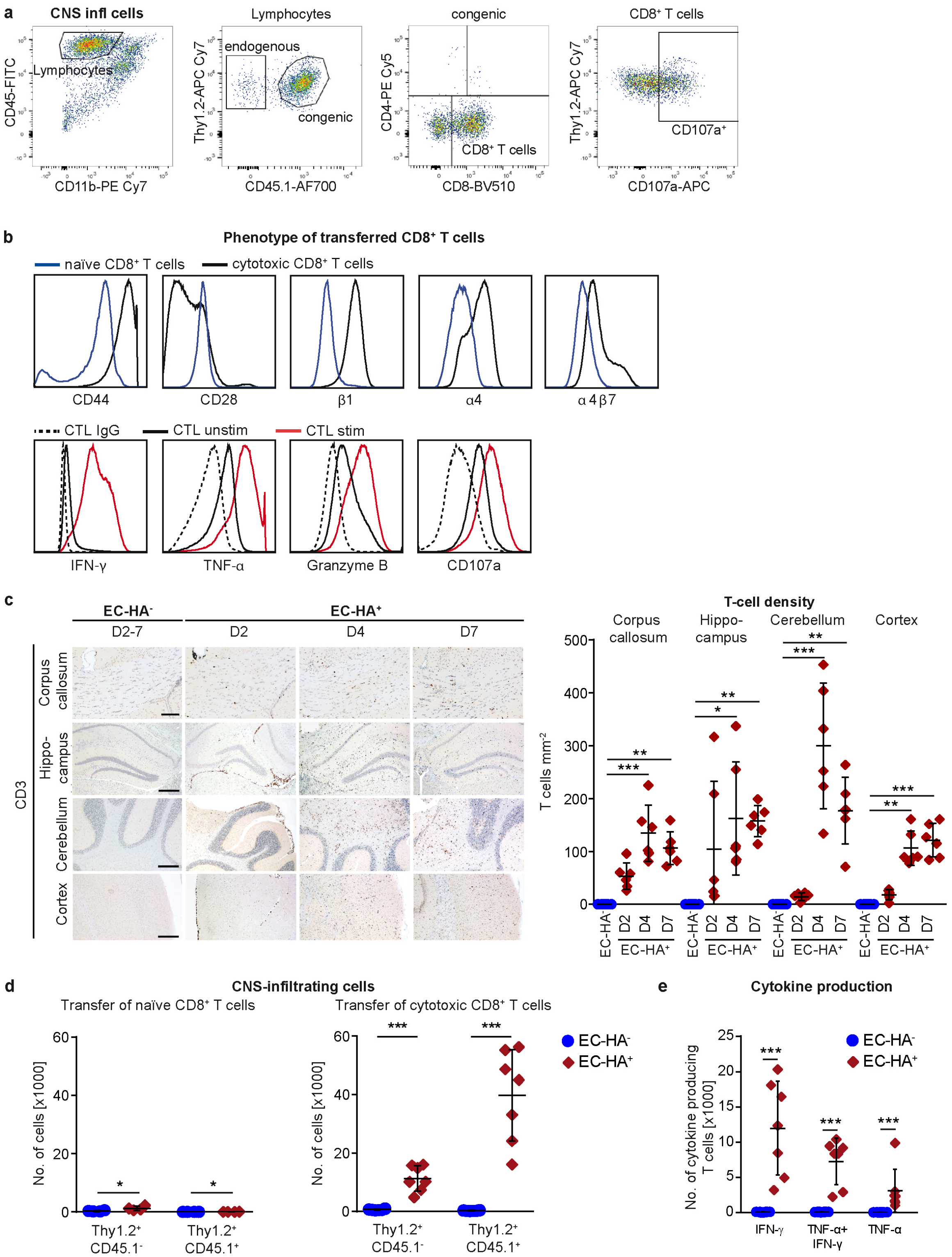
a
CNS infl cells
Lymphocytes
CD45-FITC
CD11b-PE Cy7
Lymphocytes
endogenous
congenic
Thy1.2-APC Cy7
CD45.1-AF700
congenic
CD4-PE Cy5
CD8-BV510
CD8⁺ T cells
CD8⁺ T cells
CD107a⁺
Thy1.2-APC Cy7
CD107a-APC
b
Phenotype of transferred CD8⁺ T cells
naïve CD8⁺ T cells
cytotoxic CD8⁺ T cells
CD44
CD28
β1
α4
α4β7
CTL IgG
CTL unstim
CTL stim
IFN-γ
TNF-α
Granzyme B
CD107a
c
EC-HA⁻
EC-HA⁺
D2-7
D2
D4
D7
CD3
Corpus callosum
Hippo-campus
Cerebellum
Cortex
T-cell density
Corpus callosum
Hippo-campus
Cerebellum
Cortex
T cells mm⁻²
EC-HA⁻
D2
D4
D7
EC-HA⁺
d
CNS-infiltrating cells
Transfer of naïve CD8⁺ T cells
Transfer of cytotoxic CD8⁺ T cells
No. of cells [x1000]
Thy1.2⁺ CD45.1⁻
Thy1.2⁺ CD45.1⁺
EC-HA⁻
EC-HA⁺
e
Cytokine production
No. of cytokine producing T cells [x1000]
IFN-γ
TNF-α+ IFN-γ
TNF-α
EC-HA⁻
EC-HA⁺

Supplementary Figure 8. Characterization of EC-HA$^+$ mice. a. Identification of CNS infiltrating (CD45$^+$CD11b$^-$) endogenous (CD45.1$^-$) and transferred (CD45.1$^+$) CD8 T cells (Thy1.2$^+$CD8$^+$) as well as their expression of CD107a as a marker for degranulation of cytolytic vesicles as shown in Figure 4B. **b.** Characterization of the HA-specific cytotoxic CD8$^+$ T cells by flow cytometry prior to their adoptive transfer. Cytotoxic CD8$^+$ T cells (black lines) and control naïve HA-specific CD8$^+$ T cells (blue lines) originating from CL4-TCR transgenic mice were analyzed for CD44, CD28, β1-integrin, α4-integrin, and α4β7-integrin expression. Data are gated on Thy1.2$^+$ CD45.1$^+$ CD8$^+$ T cells (top). Cytotoxic CD8$^+$ T cells were stimulated (red lines) or not (black lines) with phorbol 12-myristate 13-acetate/ionomycin for 4 hours. Staining for surface CD107a and intracellular IFN-γ, TNF-α, and granzyme B is shown. Labeled isotype control antibodies delineate the background staining (dashed lines). One representative experiment out of four is shown. **c.** Representative histological sections documenting T-cell infiltration (CD3$^+$, brown) in distinct regions of the brain (corpus callosum, hippocampus, cerebellum, and cortex) of EC-HA$^-$ and EC-HA$^+$ mice 2, 4 or 7 days after adoptive transfer of cytotoxic CD8$^+$ T cells (left). Scale bars 500 µm. Quantification of T-cell density in different regions of the CNS in EC-HA$^-$ (closed blue circles) or EC-HA$^+$ (cayenne diamonds) mice (right, n = 6). For EC-HA$^-$ mice, the data of the 3 time-points have been pooled. **d.** Quantification of CNS-infiltrating endogenous (Thy1.2$^+$CD45.1$^-$, left) and transferred (Thy1.2$^+$CD45.1$^+$, right) T cells on day 7 after adoptive transfer of either naïve (left graph) or cytotoxic (right graph) HA-specific CD8$^+$ T cells in CD45.2$^+$ EC-HA$^-$ (closed blue circles) or EC-HA$^+$ recipient mice (cayenne diamonds) (n = 4 – 8 per group). **e.** Absolute numbers of Thy1.2$^+$CD45.1$^+$ cells expressing IFN-γ and/or TNF-α upon *ex vivo* stimulation with PMA/ionomycin (n = 7-8 per group, 2 independent experiments). Statistical analysis was performed using the Kruskal-Wallis test with Dunn's post-test (c) or Mann-Whitney test (d, e), respectively. Error bars indicate the mean ± s.d.; *: $p < 0.05$; **: $p < 0.01$; ***: $p < 0.001$. Source data are provided as a Source Data file.

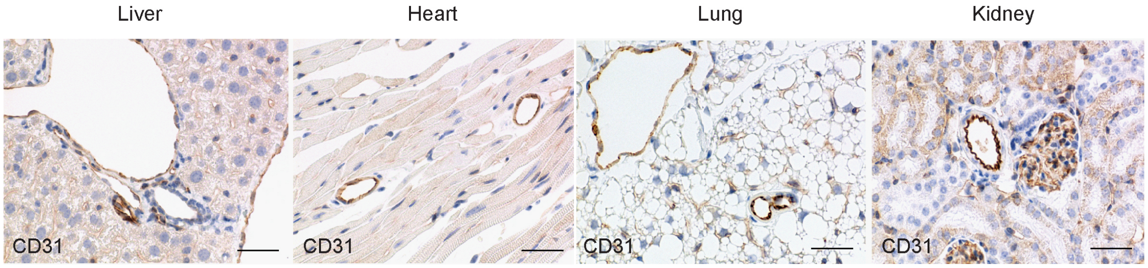

Supplementary Figure 9. Analysis of the endothelium in other organs. Representative histology sections of peripheral tissues 7 days after adoptive transfer of cytotoxic CD8 T cells in EC-HA^{+} mice. Staining of liver, heart, lung, and kidney with anti-CD31 antibodies. Data are from one representative mouse out of 3. Bar: 25µm.

Supplementary Tables

Blood/CSF samples	HD	SoD	SuS	MS
n-number	77	76	35	262
Age*1 (± SD)	41 ± 12	39 ± 17	34 ± 9	34 ± 12
Number females<br>% females	46/77<br>60%	47/76<br>62%	26/35<br>74%	189/262<br>72%
Brain involvement (1)	n.a.	n.a.	35/35	n.a.
Retinal involvement (2)	n.a.	n.a.	35/35	n.a.
Vestibulocochlear involvement (3)	n.a.	n.a.	35/35	n.a.
Definite SuS	n.a.	n.a.	35/35	n.a.
Disease stage (active)	n.a.	n.a.	13/35	169/262

Biospecimen for histology	Control*2	SuS	MS
n-number	6	7	6
Age*1 (± SD)	40 ± 6	32 ± 10	51 ± 12
Number females<br>% females	4/6<br>67%	5/7<br>71%	4/6<br>67%
Brain involvement (1)	n.a.	7/7	n.a.
Retinal involvement (2)	n.a.	5/7*3	n.a.
Vestibulocochlear involvement (3)	n.a.	7/7	n.a.
Definite SuS	n.a.	5/7	n.a.
Probable SuS	n.a.	2/7	n.a.
Disease stage (active)	n.a.	7/7	6/6

Supplementary Table 1: Patient demographics.

(1.) According to the diagnostic criteria for SuS[1], diagnostic criteria for brain involvement are fulfilled, when the patient exhibits SuS typical findings on cranial MRI such as hyperintense, multifocal, round small lesions, at least one of them in the corpus callosum (“snowball”) in T2 (or FLAIR) weighted sequences. Additionally, the patient needs to show cognitive impairment and/or focal neurological symptoms and/or headache.

(2.) The diagnostic criteria for retinal involvement are fulfilled, when the patient shows branch retinal artery occlusions (BRAO) or AWH or retinal branch ischemia or SD-OCT lesion.

(3.) The diagnostic criteria for vestibulocochlear involvement are fulfilled when the patient develops new tinnitus and/or hearing loss and/or peripheral

vertigo. The hearing loss must be confirmed by audiogram and the vestibular vertigo must be confirmed by specific diagnostics.

Patients fulfilling all three diagnostic criteria are definite-, patients fulfilling two criteria are probable-, and patient only fulfilling one criteria are possible SuS patients.

*[1] Mean age at blood / biopsy withdrawal.

*[2] Patient with no neurological disease.

*[3] No angiography was performed for one patient. Another patient had visual symptoms, but BRAO was not confirmed at the time.

HD: healthy donors, MS: multiple sclerosis, n.a.: not applicable, NIC: non-inflammatory control, SD: standard deviation, SoD: somatoform disorder, SuS: Susac syndrome.

Parameter	Reference values	SuS (n = 14)	MS (n = 222)
**Cells μl^{-1} CSF**	< 5	2.4 ± 4.6	6.8 ± 8.8
**Total protein CSF [mg ml^{-1}]**	< 500	639 ± 390	449 ± 151
**Integrity of blood-CSF barrier**	Intact	5[1]/14 (36%) with disturbance	46/222 (21%) with disturbance
**Intrathecal IgG synthesis (OCBs)**	None	0/14 (0%)	184/222 (83%)
**Intrahecal IgG synthesis (Reiber)**	None	0/14 (0%)	120/222 (54%)
**Intrathecal IgA synthesis (Reiber)**	None	0/14 (0%)	12/222 (5%)
**Intrathecal IgM synthesis (Reiber)**	None	0/14 (0%)	24/222 (11%)

Supplementary Table 2. Laboratory parameters of the cerebrospinal fluid.
CSF: cerebrospinal fluid, Q_{Gluc}: glucose quotient
[1]Two patients showed a disturbance only 3 months after the first visit.

Patient	Amino acid sequence	*Vβ* gene	*Jβ* gene	% of CD8⁺ T-cell repertoire	% of CD8⁺ T_{EMRA} repertoire
**# 4**	*CASSLEGRDSHFGANVLTF*	*TCRBV07-09*	*TCRBJ02-06*01*	4.91	13.02
	CASSPLDSNNEQFF	*TCRBV28-01*01*	*TCRBJ02-01*01*	4.95	6.03
	CATSDGLNGAGKNIQYF	*TCRBV24*	*TCRBJ02-04*01*	1.94	5.03
	CASRIGPGNNEQFF	*TCRBV27-01*01*	*TCRBJ02-01*01*	0.93	3.62
	CASSLDPRVPYNEQFF	*TCRBV07-03*01*	*TCRBJ02-01*01*	1.37	3.13
**# 5**	*CASSLLDFTDTQYF*	*TCRBV12*	*TCRBJ02-03*01*	1.04	4.73
	CAISELGTATDTQYF	*TCRBV10-03*01*	*TCRBJ02-03*01*	0.47	4.13
	CASSLELGPTREQYF	*TCRBV28-01*01*	*TCRBJ02-07*01*	0.55	3.60
	CSAKRRGQLNQPQHF	*TCRBV20*	*TCRBJ01-05*01*	1.07	2.76
	CASSDRQMRNQPQHF	*TCRBV02-01*01*	*TCRBJ01-05*01*	0.36	2.71
	CAISEPMPWSGANVLTF	*TCRBV10-03*01*	*TCRBJ02-06*01*	0.49	2.69
	CASSPHPRGQPQHF	*TCRBV27-01*01*	*TCRBJ01-05*01*	0.82	2.66
**# 10**	*CASSPPQAGANEKLFF*	*TCRBV11-02*02*	*TCRBJ01-04*01*	5.33	14.01
	CASRVFSGQGDTEAFF	*TCRBV19-01*	*TCRBJ01-01*01*	0.72	7.06
	CASSTAFDSGNTIYF	*TCRBV19-01*	*TCRBJ01-03*01*	0.33	3.12
	CASSRGQDIEETQYF	*TCRBV03*	*TCRBJ02-05*01*	0.35	2.05
	CASSQARGFSYNEQFF	*TCRBV04-03*01*	*TCRBJ02-01*01*	0.50	2.02
	CASSRGLAGYSTDTQYF	*TCRBV05-01*01*	*TCRBJ02-03*01*	0.88	1.98
	CASSIGTTSYNEQFF	*TCRBV04-01*01*	*TCRBJ02-01*01*	0.71	1.94
	CASSSDRDSVNGELFF	*TCRBV07-09*	*TCRBJ02-02*01*	0.22	1.94
**# 12**	*CASSFTAGGNIQYF*	*TCRBV06-06*	*TCRBJ02-04*01*	4.40	30.85
	CASSFQGQGINTGELFF	*TCRBV27-01*01*	*TCRBJ02-02*01*	0.73	3.83
	CASSPWELLTYSSPQLQFF	*TCRBV04-02*01*	*TCRBJ02-01*01*	1.01	3.39
	CASSFGIATSGVPQETQYF	*TCRBV28-01*01*	*TCRBJ02-05*01*	0.57	2.64
	CASSTTGVILTDTQYF	*TCRBV19-01*	*TCRBJ02-03*01*	0.55	2.38
	CASSLATRQALSYEQYF	*TCRBV27-01*01*	*TCRBJ02-07*01*	4.16	2.04
	CASKNGLAYNEQFF	*TCRBV27-01*01*	*TCRBJ02-01*01*	0.24	1.32
**# 25**	*CASSLASQAGEKLFF*	*TCRBV07-09*	*TCRBJ01-04*01*	1.98	12.32
	CASSRLAATNTGELFF	*TCRBV07-03*01*	*TCRBJ02-02*01*	1.66	8.04
	CASSQGELAGGSYEQYF	*TCRBV14-01*01*	*TCRBJ02-07*01*	2.99	5.43
	CASSVTRDRGNTGELFF	*TCRBV09-01*	*TCRBJ02-02*01*	1.83	5.20
	CSVVGTGFSYEQYF	*TCRBV29-01*01*	*TCRBJ02-07*01*	3.73	4.11
	CATSPGNTDTQYF	*TCRBV28-01*01*	*TCRBJ02-03*01*	1.31	3.30
	CASSRREDRPTGELFF	*TCRBV07-09*	*TCRBJ02-02*01*	0.54	3.27
	CASSVARDRGNTGELFF	*TCRBV09-01*	*TCRBJ02-02*01*	0.81	3.21
**# 30**	*CASSLVLGTGGLQAYEQYF*	*TCRBV07-02*01*	*TCRBJ02-07*01*	2.09	10.27
	CASSQDGVWDEQFF	*TCRBV04-03*01*	*TCRBJ02-01*01*	0.84	8.65
	CATSDSDRWKQFF	*TCRBV24*	*TCRBJ02-01*01*	4.42	3.13
	CASSWSMGRRDEQYV	*TCRBV13-01*01*	*TCRBJ02-07*01*	0.57	2.80
	CASSSPFTAGGVQETQYF	*TCRBV28-01*01*	*TCRBJ02-05*01*	0.64	2.26
	CASSFPIVEGPSTDTQYF	*TCRBV28-01*01*	*TCRBJ02-03*01*	0.36	2.08
	CASSLRGEGSPAAEQFF	*TCRBV07-09*	*TCRBJ02-01*01*	1.10	1.84
	CASSLEYHTF	*TCRBV13-01*01*	*TCRBJ01-02*01*	0.38	1.40

Supplementary Table 3. SuS-specific private CD8⁺ T-cell and CD8⁺ T_{EMRA} clones. Table summarizing the patient-specific private clones, their *Vβ*-gene and *Jβ*-gene usage, and their frequency in the CD8⁺ T-cell repertoire and CD8⁺ T_{EMRA} repertoire of the SuS patients.

Patient	4	5	8	9	10	11	12	14	16	21	22	25	27	30
HLA-A	**02*	**01*	**02*	**02*	**02*	**02*	**24*	**03*	**02*	**02*	**01*	**01*	**11*	**02*
	**02*	**69*	**02*	**68*	**03*	**03*	**68*	**30*	**36*	**02*	**24*	**01*	**30*	**03*
HLA-B	**07*	**55*	**07*	**35*	**07*	**07*	**40*	**07*	**08*	**51*	**15*	**08*	**13*	**07*
	**57*	**57*	**40*	**35*	**40*	**51*	**44*	**07*	**15*	**58*	**57*	**13*	**58*	**40*
HLA-C	**06*	**01*	**07*	**04*	**03*	**01*	**03*	**05*	**03*	**02*	**06*	**06*	**06*	**03*
	**07*	**06*	**07*	**04*	**07*	**07*	**07*	**07*	**07*	**07*	**07*	**07*	**07*	**07*

Supplementary Table 4. HLA class-I typing of SuS-patients. Allele frequencies of *HLA-A*02*, *HLA-B*07* and *HLA-C*07* in SuS patients were 0.3929, 0.2500, and 0.4643, respectively. Allele frequencies of *HLA-A*02*, *HLA-B*07* and *HLA-C*07* in control cohort of 39689 German, Caucasian individuals http://allelfrequencies.net are 0.2826, 0.1253, and 0.3008, respectively.

Antigen	Fluorochrome	Clone	Manufacturer	Reactivity
**Antibodies for flow cytometry**				
**CD3**	FITC	UCHT1	Biolegend	Human
**CD3**	PerCP/Cy5.5	OKT3	Biolegend	Human
**CD3**	PerCP/Cy5.5	UCHT1	Biolegend	Human
**CD3**	PC5.5	UCHT1	Beckman Coulter	Human
**CD3**	BV510	UCHT1	Biolegend	Human
**CD4**	FITC	OKT4	Biolegend	Human
**CD4**	APC	OKT4	Biolegend	Human
**CD4**	APC	13B8.2	Beckman Coulter	Human
**CD4**	BV421	OKT4	Biolegend	Human
**CD4**	BV510	OKT4	Biolegend	Human
**CD8**	FITC	RPA-T8	Biolegend	Human
**CD8**	ECD	2ST8.5H7	Beckman Coulter	Human
**CD8**	APC	B9.11	Beckman Coulter	Human
**CD8**	PB	HIT8a	Biolegend	Human
**CD8**	PB	B9.11	Beckman Coulter	Human
**CD14**	FITC	RM052	Beckman Coulter	Human
**CD14**	BV510	M5E2	Biolegend	Human
**CD16**	AF750	3G8	Beckman Coulter	Human
**CD19**	AF700	HIBI9	Biolegend	Human
**CD19**	AF700	J3-119	Beckman Coulter	Human
**CD25**	APC	B1.49.9	Beckman Coulter	Human
**CD27**	PC7	1A4CD27	Beckman Coulter	Human
**CD45**	KrO	J.33	Beckman Coulter	Human
**CD45RA**	FITC	ALB11	Beckman Coulter	Human
**CD45RA**	APC	H100	Biolegend	Human
**CD56**	PE	N901	Beckman Coulter	Human
**CD56**	APC	HCD56	Biolegend	Human
**CD56**	PC7	N901	Beckman Coulter	Human
**CD62L**	APC	DREG-56	Biolegend	Human
**CD62L**	AF750	DREG-56	Biolegend	Human
**CD107a**	AF488	ID4B	Biolegend	Human
**CD127**	AF700	A019D5	Biolegend	Human
**CD138**	PE	B-A38	Beckman Coulter	Human
**Foxp3**	PE	PCH101	eBioscience	Human
**Granzyme B**	AF700	GB11	Biolegend	Human
**HLA-DR**	ECD	Immu-357	Beckman Coulter	Human
**Perforin**	PE	dG9	Biolegend	Human
**Perforin**	PB	B-D48	Beckman Coulter	Human
**α4β7**	PE	DATK32	eBioscience	Mouse
**β1-integrin**	FITC	Ha215	BD Biosciences	Mouse
**CD4**	PeCy5	RM4-5	BD Biosciences	Mouse
**CD4**	BV421	RM4-5	BD Biosciences	Mouse
**CD4**	BV510	RM4-5	eBioscience	Mouse
**CD8α**	BV510	53-6.7	BD Pharmingen	Mouse
**CD8α**	BV650	53-6.7	BD Pharmingen	Mouse
**CD11b**	PeCy7	M1/70	eBioscience	Mouse

**CD11c**	PerCP/Cy5.5	N418	eBioscience	Mouse
**CD25**	A700	PC61	BD Biosciences	Mouse
**CD28**	PerCpCy5.5	37.51	ThermoFisher	Mouse
**CD44**	PeCy5	IM7	eBioscience	Mouse
**CD45**	FITC	30-F11	BD Biosciences	Mouse
**CD45.1**	PerCP/Cy5.5	A20	eBioscience	Mouse
**CD45.1**	A700	A20	Biolegend	Mouse
**CD49d**	BV786	R1-2	BD Biosciences	Mouse
**CD62L**	FITC	MEL-14	BD Biosciences	Mouse
**CD62L**	APC	MEL-14	BD Biosciences	Mouse
**CD69**	APC	H1.2F3	BD Biosciences	Mouse
**CD107a**	APC	1D4B	Biolegend	Mouse
**Granzyme B**	PE	NGZB	eBioscience	Mouse
**Interferon-γ**	BV421	XMG.1	BD Biosciences	Mouse
**MHC class-II**	BV711	M5/114.15.2	eBioscience	Mouse
**Thy1.2**	APC-Cy7	53-2.1	eBioscience	Mouse
**TNF-α**	BV650	MP6-XT22	BD Biosciences	Mouse
**Antibodies for immunohistochemistry and fluorescence microscopy**				
**APP**		MAB348	Merck/Millipore	Human/ Mouse
**Caspase-3**	unconjugated	#9664	Cellsignal	Human/ Mouse
**CD3**	unconjugated	SP7	BD	Human/ Mouse
**CD4**	unconjugated	4B12	DAKO	Human
**CD8 (fluorescence)**	unconjugated	SP16	Thermo Scientific	Human
**CD8 (IHC)**	unconjugated	M7103	DAKO	Human
**CD20**	unconjugated	MS-340	Thermo Scientific	Human
**CD31**	unconjugated	JC70	BD	Human
**CD34**	unconjugated	NCL-L-END	Novocastra	Human
**CD138**	unconjugated	MCA681H	Serotec	Human
**GFAP**	unconjugated	20334	DAKO	Human
**Granzyme B**	unconjugated	MS-1157	Thermo Scientific	Human
**MBP**	unconjugated	A0623	DAKO	Human
**MHC Class I**	unconjugated	HC10	Gift from Dr. Ploegh, Harvard, USA	Human
**Carbonic Anhydrase II**		PC076	the Binding Site	Mouse
**Beta-2-Microglobulin**	unconjugated	M-20	Santa Cruz	Mouse
**CD8α**	unconjugated	4SM15	eBioscience	Mouse
**CD31**	unconjugated	ab28364	Abcam	Mouse
**CD34**	unconjucated	SM1603P	Acris	Mouse
**GFAP**	unconjugated	814369	Boehringer	Mouse
**Granzyme B**	unconjugated	ab4059	Abcam	Mouse

Supplementary Table 5. Antibodies used during the study. AF: Alexa Fluor, APC: Allophycocyanin, BV: Brilliant Violet, ECD: Electron coupled dye, FITC: Fluorescein isothiocyanate, PE: Phycoerythrin, PerCP: Peridinin-chlorophyll-protein Complex Conjugate, KrO: Krome orange.

References

1. Kleffner I, Dörr J, Ringelstein M, Gross CC, Böckenfeld Y, Schwindt W, *et al.* Diagnostic criteria for Susac syndrome. *J Neurol Neurosurg Psychiatry* 2016.
