# Supplementary material for: CD8+ T cell-mediated endotheliopathy is a targetable mechanism of neuro-inflammation in Susac syndrome
Source: Nat Commun. 2019 Dec 18;10:5779. doi: 10.1038/s41467-019-13593-5 (PMC6920411; doi:10.1038/s41467-019-13593-5)
Supplement: Supplementary file 3 — Reporting Summary [file 41467_2019_13593_MOESM3_ESM.pdf]

nature research

Corresponding author(s): Heinz Wiendl
Roland Liblau
Catharina C. Gross
NCOMMS-19-28933-T

Last updated by author(s): Oct 28, 2019

Reporting Summary

Nature Research wishes to improve the reproducibility of the work that we publish. This form provides structure for consistency and transparency in reporting. For further information on Nature Research policies, see Authors & Referees and the Editorial Policy Checklist.

Statistics

For all statistical analyses, confirm that the following items are present in the figure legend, table legend, main text, or Methods section.

n/a	Confirmed	
☐	☒	The exact sample size (*n*) for each experimental group/condition, given as a discrete number and unit of measurement
☐	☒	A statement on whether measurements were taken from distinct samples or whether the same sample was measured repeatedly
☐	☒	The statistical test(s) used AND whether they are one- or two-sided<br>*Only common tests should be described solely by name; describe more complex techniques in the Methods section.*
☐	☒	A description of all covariates tested
☐	☒	A description of any assumptions or corrections, such as tests of normality and adjustment for multiple comparisons
☐	☒	A full description of the statistical parameters including central tendency (e.g. means) or other basic estimates (e.g. regression coefficient) AND variation (e.g. standard deviation) or associated estimates of uncertainty (e.g. confidence intervals)
☐	☒	For null hypothesis testing, the test statistic (e.g. *F*, *t*, *r*) with confidence intervals, effect sizes, degrees of freedom and *P* value noted<br>*Give P values as exact values whenever suitable.*
☒	☐	For Bayesian analysis, information on the choice of priors and Markov chain Monte Carlo settings
☒	☐	For hierarchical and complex designs, identification of the appropriate level for tests and full reporting of outcomes
☐	☒	Estimates of effect sizes (e.g. Cohen's *d*, Pearson's *r*), indicating how they were calculated

Our web collection on statistics for biologists contains articles on many of the points above.

Software and code

Policy information about availability of computer code

Data collection	Human flow cytometry data were collected using the using Kaluza Acquisition software on the Gallios / Navios (latter was used for CSF flow cytometry) (both Beckman Coulter). Murine flow cytometry data were collected on BD Fortessa using the software BD FACSDiva.<br>Adhesion of CD8 T cells was recorded using the BZ-900 BioRevo and BZ II viewer software (Keyence)<br>Immunofluorescence stainings were examined using a confocal laser scan microscope (Leica SP5)<br>TCR sequencing was performed using ImmunoSeq Platform Technology (Adaptive Biotechnologies) and the Illumina HiSeq Platform
Data analysis	Data and statistical analyses were performed using GraphPad Prism v5.0.<br>Microsoft Excel 2010, immuneSEQ Analyzer v2.0 (Adaptive Biotechnologies), immuneACCESS (Adaptive Biotechnologies), and GeneMarker Software (Softgenetics) for TCR sequencing and spectratyping.<br>Flow cytometry data were analyzed by Kaluza Analysis software 1.5a (Beckman Coulter, human data) and FlowJo version 10.4 software (LLC, mouse data).<br>Histology data were analyzed by ImageJ (NIH)

For manuscripts utilizing custom algorithms or software that are central to the research but not yet described in published literature, software must be made available to editors/reviewers. We strongly encourage code deposition in a community repository (e.g. GitHub). See the Nature Research guidelines for submitting code & software for further information.

Data

Policy information about availability of data

All manuscripts must include a data availability statement. This statement should provide the following information, where applicable:

- Accession codes, unique identifiers, or web links for publicly available datasets
- A list of figures that have associated raw data
- A description of any restrictions on data availability

There is no restriction in the availability of materials described in the study. Data are available on request from the corresponding authors.

Field-specific reporting

Please select the one below that is the best fit for your research. If you are not sure, read the appropriate sections before making your selection.

☒ Life sciences ☐ Behavioural & social sciences ☐ Ecological, evolutionary & environmental sciences

For a reference copy of the document with all sections, see nature.com/documents/nr-reporting-summary-flat.pdf

Life sciences study design

All studies must disclose on these points even when the disclosure is negative.

Sample size	Given the rarity of the disease all Susac syndrome patients (42) who consented to participate in the study were considered. Furthermore we used age- and sex matched healthy individuals (77), patients with somatoform disorders (76), and treatment naive multiple sclerosis patients (262) as controls.<br>For the murine study sample sizes were chosen to ensure adequate power with the statistical tests while minimizing the number of animals used in compliance with ethical guidelines.
Data exclusions	Since some of the experiments were already performed prior to the diagnosis being finalized, patients that did not fulfill the diagnostic criteria for Susac syndrome (Kleffner et al., 2016, J Neurol Neurosrug Psychiatry) and revised McDonald criteria for multiple sclerosis were excluded.<br>For one mouse out of 134 mice used for the study (53 EC-HA- and 81 EC-HA+) the genotype was ambiguous and tissue/DNA not available for re-assessment. This mouse therefore was removed from the analysis.
Replication	For the human study, every patient and control individual was treated as a biological replicate.<br>Murine experimental findings were reliably reproduced in at least two independent experiments except when specifically indicated.
Randomization	Since this was an exploratory study no randomization was not performed for the human part of the study.<br>Mice of both sexes were randomized into the different groups.
Blinding	For the evaluation of mouse motor performance and weight recording, the technicians performing injections into mice, taking the weight and performing the rotarod analysis were blinded to the experimental groups or genotype of the animals.

Reporting for specific materials, systems and methods

We require information from authors about some types of materials, experimental systems and methods used in many studies. Here, indicate whether each material, system or method listed is relevant to your study. If you are not sure if a list item applies to your research, read the appropriate section before selecting a response.

Materials & experimental systems

n/a	Involved in the study
☐	☒ Antibodies
☐	☒ Eukaryotic cell lines
☒	☐ Palaeontology
☐	☒ Animals and other organisms
☐	☒ Human research participants
☐	☒ Clinical data

Methods

n/a	Involved in the study
☒	☐ ChIP-seq
☐	☒ Flow cytometry
☐	☒ MRI-based neuroimaging

Antibodies

Antibodies used	Flow cytometry antibody reagents listed below in the following order:<br>Protein/Fluorochrome/Clone/Manufacturer/Animal/Lot Number/Reference number<br>CD3 PerCP/Cy5.5 UCHT1 Biolegend Human B240526 300430<br>CD3 PerCP/Cy5.5 OKT3 Biolegend Human B186624,B231920 317336<br>CD3 PC5.5 UCHT1 Beckman Coulter Human A66327 23,25,26

CD3 FITC OKT3 Biolegend Human B143565 300306
CD3 BV510 OKT3 Biolegend Human B246540 317332
CD4 FITC RPA-T4 Biolegend Human B225958 300506
CD4 BV421 OKT4 Biolegend Human B227581 317434
CD4 BV510 OKT4 Biolegend Human B235088 317444
CD4 APC 13B8.2 Beckman Coulter Human 40,43 IM2468
CD8 APC B9.11 Beckman Coulter Human 42 IM2469
CD8 Pacific Blue HIT8a Biolegend Human B209345 300928
CD8 FITC RPA-T8 Biolegend Human B153608 301006
CD8 ECD 2ST8.5H7 Beckman Coulter Human 7974052 737659
CD8 Pacific Blue B9.11 Beckman Coulter Human 20,23,25,27 A82791
CD14 FITC RM052 Beckman Coulter Human 1,2,3,5 B36297
CD14 BV510 M5E2 Biolegend Human B187862 301841
CD16 AF750 3G8 Beckman Coulter Human 29,30,31,34 A66330
CD19 AF700 J3-119 Beckman Coulter Human 1,3,4,5,25,26,27 B49212
CD25 APC B1.49.9 Beckman Coulter Human 08 B09684
CD27 PeCy7 1A4CD27 Beckman Coulter Human 22 A54823
CD45 KrO J.33 Beckman Coulter Human 6,8,10,12,19 B36294
CD45RA FITC ALB11 Beckman Coulter Human 20 A07786
CD45RA APC H100 Biolegend Human B238560 304112
CD56 PE N901 Beckman Coulter Human 43,44,45,48 A21692
CD56 APC HCD56 Biolegend Human B210225, B194250 318310
CD56 PeCy7 N901 Beckman Coulter Human 41 A21692
CD62L APC DREG-56 Biolegend Human B192564 304810
CD62L AF750 DREG-56 Invitrogen Human MHCD62L27 1746668A
CD107a AF488 H4A3 Biolegend Human B214155 328610
CD127 AF700 R34.34 Beckman Coulter Human 13 A71116
CD138 PE B-A38 Beckman Coulter Human 15,20 A54190
Foxp3 PE PCH101 eBioscience Human E09963-1634, E09963-1636 12-4776-73
Granzyme B AF700 GB11 BD Pharmingen Human 5306719 560213
HLA-DR ECD Immu-357 Beckman Coulter Human 49,52,53,54,56 IM36336
Perforin Pacific Blue B-D48 Beckman Coulter Human 4 B46030
Perforin PE dG9 Biolegend Human B150156, B195260 308106
α4β7 PE DATK32 eBioscience Mouse E01882-1633 12-5887-82
β1-integrin FITC Ha215 BD Biosciences Mouse 3350527 561796
CD4 PeCy5 RM4-5 BD Biosciences Mouse B209403 100514
CD4 BV421 RM4-5 BD Biosciences Mouse 5314582 558107
CD4 BV510 RM4-5 eBioscience Mouse 7137974 563106
CD8α BV510 53-6.7 BD Pharmingen Mouse B245634 100752
CD8α BV650 53-6.7 BD Pharmingen Mouse 7047617 563234
CD11b PeCy7 M1/70 eBioscience Mouse 7033964 552850
CD11c PerCpCy5.5 N418 eBioscience Mouse B212000 117328
CD25 AF700 PC61 BD Biosciences Mouse 70485583 565135
CD28 PerCpCy5.5 37.51 ThermoFisher Mouse 5314582 558107
CD44 PeCy5 IM7 eBioscience Mouse 4312993 15-0441-81
CD45 FITC BB515 30-F11 BD Biosciences Mouse 7339576 564590
CD45.1 PerCP/Cy5.5 A20 eBioscience Mouse E08340-1635 45-0453-82
CD45.1 AF700 A20 Biolegend Mouse B202280 110724
CD49d BV786 R1-2 BD Biosciences Mouse 7117657 564397
CD62L FITC MEL-14 BD Biosciences Mouse 5090952 553150
CD62L APC MEL-14 BD Biosciences Mouse 3035949 553152
CD69 APC H1.2F3 BD Biosciences Mouse 6005671 560689
CD107a APC 1D4B Biolegend Mouse 7082925 560646
Granzyme B PE NGZB eBioscience Mouse 4322552 12-8898-82
Interferon-g BV421 XMG.1 BD Biosciences Mouse 7135887 563376
MHC class-II BV711 M5/114.15.2 eBioscience Mouse B240003 107643
Thy1.2 APC-Cy7 53-2.1 eBioscience Mouse 7061947 561641
TNF-α BV650 MP6-XT22 BD Biosciences Mouse 7117629 563943
CD3 (unconjugated) SP7 Thermo Scientific Mouse/Human MA1-90582
CD4 (unconjugated) 4B12 DAKO Human M7310
CD8 (fluorescence) (unconjugated) SP16 Thermo Scientific Human 9116S1310G MA5-14548
CD8a (immunehistoch.) (unconjugated) C8/144B DAKO Human M7103
CD8a (unconjugated) 4SM15 eBioscience Mouse 14-0808
CD20 (unconjugated) L26 Thermo Scientific Human MS-340
CD138 (unconjugated) MCA681H Serotec Human 280599 B-A38
Granzyme B (unconjugated) polyclonal Abcam Mouse 1157S403A ab4059
Granzyme B (unconjugated) GZB01 Thermo Scientific Human MS-1157
Beta-2-Microglobulin (unconjugated) polyclonal Santa Cruz Mouse sc-8361
MHC Class I (unconjugated) HC10 Human (Gift from Dr. Ploegh, Harvard, USA; Stam, N.J., Vroom, T.M., Peters, P.J., Pastoors, E.B. & Ploegh, H.L. HLA-A- and HLA-B-specific monoclonal antibodies reactive with free heavy chains in western blots, in formalin-fixed, paraffin-embedded tissue sections and in cryo-immuno-electron microscopy. Int Immunol 2, 113-125 (1990).)
Caspase-3 (unconjugated) 5A1 Cellsignal Mouse/Human 21 9664
CD31 (unconjugated) MEC 13.1 BD Mouse 550274
CD31 (unconjugated) polyclonal Abcam Mouse ab28364
GFAP (unconjugated) polyclonal DAKO Human 96 Z0334

	GFAP (unconjugated) G-A-5 Millipore/Boehringer Mouse 14119823 MAB3402<br>MBP (unconjugated) polyclonal DAKO Human 108 A0623<br>Carbonic Anhydrase II (unconjugated) polyclonal the Binding Site Mouse 703111 PC076<br>APP (unconjugated) 22C11 Merck/Millipore Mouse/Human 2987447 MAB348
Validation	Antibodies purchased were validated by the manufacturer. Antibodies used for routine CSF flow cytometry were also tested daily by staining of ImmunTrol (BeckmanCoulter, 6607077) reference samples. Briefly, ImmunoTrol cells are processed as described for blood and CSF samples and analyzed by flow cytometry.<br>Performance of flow cytometers was tested prior to acquisition by FlowCheck Pro Fluorospheres (BeckmanCoulter, A63493).

Eukaryotic cell lines

Policy information about cell lines

Cell line source(s)	P815 cells were acquired from ATCC TIB-64
Authentication	Cell line was authenicated by the manufacturer.
Mycoplasma contamination	Cell line was not tested for Mycoplasma contamination.
Commonly misidentified lines<br>(See ICLAC register)	Not applicable

Animals and other organisms

Policy information about studies involving animals; ARRIVE guidelines recommended for reporting animal research

Laboratory animals	All procedures were performed in male and female (BALB/cxC57Bl/6)F1 mice between 6 and 20 weeks of age
Wild animals	Not applicable
Field-collected samples	Not applicable
Ethics oversight	Université Toulouse registration no. 16-U1043 RL/CM-653

Note that full information on the approval of the study protocol must also be provided in the manuscript.

Human research participants

Policy information about studies involving human research participants

Population characteristics	42 patients with SuS according to the recently defined diagnostic criteria were included in this study. 262 treatment-naïve patients with a stable RRMS fulfilling the revised McDonald criteria were included in the study. Peripheral blood mononuclear cells (PBMCs) from 77 age-and sex-matched healthy donors served as controls. 76 individuals with somatoform disease also served as controls for CSF analysis. The average age of cohorts at the time of performing the experiment are: 41 ± 12 (HDs), 39 ± 17 (SoD), 34 ± 9 (SuS) and 34 ± 12 (MS) respectively. The % of female individuals in each cohort are 60% (HD), 62% (SoD), 74% (SuS) and 72% (MS) respectively.
Recruitment	All studies and clinical investigations were conducted according to the Declaration of Helsinki and approved by the ethic committee of the University of Münster; registration nos. 2010-262-f-S, 2011-665-f-S and 2014-068-f-S, 2012-407-f-S, the ethics committee of the Medical University of Vienna; registration nos. 1206/2013, 1123/2015, and the Human Research Ethics Committee of the Royal Prince Alfred Hospital in Sydney, Australia; approval numbers X11/0202 & X09/0371. All patients provided written formal consent before participating in the study. Lumbar puncture and CNS biopsy were only performed for diagnostic reasons.
Ethics oversight	Ethic committee of the University of Münster; registration nos. 2010-262-f-S, 2011-665-f-S and 2014-068-f-S, 2012-407-f-S and the ethics committee of the Medical University of Vienna; registration nos. 1206/2013, 1123/2015.

Note that full information on the approval of the study protocol must also be provided in the manuscript.

Clinical data

Policy information about clinical studies

All manuscripts should comply with the ICMJE guidelines for publication of clinical research and a completed CONSORT checklist must be included with all submissions.

Clinical trial registration	not applicable
Study protocol	not applicable
Data collection	not applicable

Outcomes

not applicable

Flow Cytometry

Plots

Confirm that:

☒ The axis labels state the marker and fluorochrome used (e.g. CD4-FITC).

☒ The axis scales are clearly visible. Include numbers along axes only for bottom left plot of group (a 'group' is an analysis of identical markers).

☒ All plots are contour plots with outliers or pseudocolor plots.

☒ A numerical value for number of cells or percentage (with statistics) is provided.

Methodology

Sample preparation

Human blood was collected in EDTA-containing tubes (K2E Vacutainer, BD) and processed within 24 hours. For blood and CSF analysis, 100 µl of peripheral blood and cerebrospinal fluid was treated with VersaLyse buffer (Beckman Coulter). Addition of FlowCount FLuorspheres allowed quantification of total cell counts. PBMCs were isolated by density gradient centrifugation using lymphocyte separation medium (PAA laboratories). For frozen PBMCs cells were thawed. For surface staining, cells were resuspended in flow cytometry buffer (PBS/0.5%BSA/2mM EDTA) containing fluorochrome-conjugated monoclonal antibodies at 4°C for 30 minutes. Cells were washed and acquired on a flow cytometer. For the identification of Granzyme B and perforin expression, surface-labeled cells were permeabilized with fixation/permeabilization solutions (BD Cytofix/Cytoperm™) followed by intracellular staining with fluorescence-labeled mAbs for the proteins.
For purification of mouse mononuclear cells, mice were anesthetized with ketamine (100 mg/kg) and xylazine (10 mg/kg) and perfused intracardially with PBS. Spleen was collected and dissociated, and red blood cells were lysed. Brains were removed and homogenized, followed by digestion with collagenase D (1 mg/mL), DNase I (10 mg/mL), and TLCK (0.02 mg/ml), and CNS-infiltrating mononuclear cells were then isolated using Percoll density separation, as described (Gebauer et al. 2017 Oncoimmunology). HA-specific CTLs were generated from CL4-TCR mice, as described (Saxena et al. 2008 J Immunol, Bernard-Valnet et al. 2016 PNAS).

Instrument

Navios and Gallios (10C3L, Beckman Coulter, human data)
LSR-Fortessa Special Order Research Product (BD Biosciences, murine data)

Software

Kaluza acquisition software and Kaluza analysis software v.1.5a (Beckman Coulter) were used to acquire and analyze human data, respectively.
To collect murine data, we used the software BD FACSDiva and to analyze the data the software FlowJo v.10.4 was used.

Cell population abundance

The abundance of isolated cells was samples was assessed with flow cytometry noting an increase in the presence of the cell type of interest and the absence of other cell types. On an average, purity of >90% of sorted cells was obtained.

Gating strategy

Gating strategies for human study:
Leukocytes were selected from total events based on FSC vs CD45 characteristics. Lymphocytes were distingusihed from monocytes and granulocytes in a SSC vs CD14 plot by gating on CD14-SSClow cells. Lymphocytes were further classified into CD19highCD138- B cells and CD19lowCD138high plasma cells in a CD19 vs CD138 plot. T cells were selected as CD3+CD56- lymphocytes from a CD3 vs CD56 plot and futher divided into CD4+CD8- and CD4-CD8+ T cells based on the respective marker expression. These cells were analyzed for HLA-DR expression in a CD3 vs HLA-DR plot.
For human CD8+ T-cell analyses, live, singlet lymphocytes were selected based on cell size and complexity (FSC/SSC). CD56+ cells were excluded and CD3+ cells were included. On CD3+CD56- cells, CD8+ cells were gated with the exclusion of CD4+ cells. Gating on these CD8+ T cells, subsets and cells expressing molecules of interest were gated on such as naive/memory cells (naive: CD45RA+CD62L+, central memory: CD45RA-CD62L+, effector memory: CD45RA-CD62L- and CD45RA-expressing effector memory: CD45RA+CD62L-), granzyme B+, perforin+ and CD107a+ expressing cells.
For the assessment of allogenic suppression assay, CD8+ and CD4+ T cells were selected as explained and proliferating cells were identified based on tracking cells labeled with efluor670 dye. CD4+ T cells were gated on as described and based on CD25 high and CD127low staining, regulatory T cells were identified.
Gating strategies for murine study:
FSC-A SSC-A was used to gate on cells. FSC-H/FSC-A and SSC-H SSC-A were used to gate on singlet cells. The T cells were gated on CD11b-CD45+Thy1.2+ cells. The congenic T cells were gated on CD11b-CD45+Thy1.2+CD45.1+ cells and the endogenous T cells were gated on CD11b-CD45+Thy1.2+CD45.1- cells. Congenic CD8+ T cells were gated as CD11b-CD45+Thy1.2+CD45.1+CD4-CD8+ cells and endogenous CD8+ T cells were gated as CD11b-CD45+Thy1.2+CD45.1-CD4-CD8+ cells. IFNg+, TNFa+, and CD107a+ cells were gated on congenic CD8+ T cells.
For the phenotyping of transferred CD8+ T cells (supplementary data 4c), FSC-A SSC-A was used to gate on the cells. FSC-H/FSC-A and SSC-H SSC-A were used to gate on the singlet cells. Congenic CD8+ T cells were gated on Thy1.2+CD45.1+CD4-CD8+ cells. CD44+, CD28+, B1+, a4 integrin+, a4b7+, IFNg+, TNFa+, Granzyme B+ and CD107a+ were gated on congenic CD8+ T cells.
A supplementary figure demonstrating gating strategies of major experiments will be included.

☒ Tick this box to confirm that a figure exemplifying the gating strategy is provided in the Supplementary Information.

Magnetic resonance imaging

Experimental design

Design type	Not applicable
Design specifications	Not applicable
Behavioral performance measures	Not applicable

Acquisition

Imaging type(s)	Structural, Diffusion (acquired, but not shown)
Field strength	1.5
Sequence & imaging parameters	2014: Philips Intera 1.5T, FLAIR sequence, 100% FOV, 256x256 matrix, slice thickness 5mm, inversion time 2.500 ms, sagittal, TE 120 ms, TR 8s, 90° flip angle<br>2015: Philips Achieva 1.5T, FLAIR sequence, 100% FOV, 256x256 matrix, slice thickness 5mm, inversion time 2.500 ms, sagittal, TE 120 ms, TR 8s, 90° flip angle
Area of acquisition	Whole Brain

Diffusion MRI ☐ Used ☒ Not used

Preprocessing

Preprocessing software	Not applicable
Normalization	Not applicable
Normalization template	Not applicable
Noise and artifact removal	Not applicable
Volume censoring	Not applicable

Statistical modeling & inference

Model type and settings	Not applicable
Effect(s) tested	Not applicable

Specify type of analysis: ☐ Whole brain ☐ ROI-based ☐ Both

Statistic type for inference (See Eklund et al. 2016)	Not applicable
Correction	Not applicable

Models & analysis

n/a	Involved in the study
☒	☐ Functional and/or effective connectivity
☒	☐ Graph analysis
☒	☐ Multivariate modeling or predictive analysis
